# Integrating Rehabilitation Services into Routine Care of Rheumatoid Arthritis May Reduce the Inflammatory Response: A Hospital-Based Follow-Up Study in Taiwan

**DOI:** 10.3390/medicina60121938

**Published:** 2024-11-25

**Authors:** Hui-Ju Huang, Wei-Jen Chen, Hanoch Livneh, Hua-Lung Huang, Ming-Chi Lu, Tzung-Yi Tsai

**Affiliations:** 1 Department of Nursing, Dalin Tzu Chi Hospital, Buddhist Tzu Chi Medical Foundation, Chiayi 62247, Taiwan; df778383@tzuchi.com.tw; 2 Center of Sports Medicine, Dalin Tzu Chi Hospital, Buddhist Tzu Chi Medical Foundation, Chiayi 62247, Taiwan; tough2915@hotmail.com; 3 Graduate Institute of Sports Science, National Taiwan Sport University, Taoyuan 333325, Taiwan; 4 School of Post-Baccalaureate Chinese Medicine, Tzu Chi University, Hualien 970374, Taiwan; 5 Rehabilitation Counseling Program, Portland State University, Portland, OR 97207-0751, USA; livnehh@pdx.edu; 6 Department of Rehabilitation, Dalin Tzu Chi Hospital, Buddhist Tzu Chi Medical Foundation, Chiayi 62247, Taiwan; 7 School of Medicine, Tzu Chi University, Hualien 97004, Taiwan; 8 Division of Allergy, Immunology and Rheumatology, Dalin Tzu Chi Hospital, Buddhist Tzu Chi Medical Foundation, Dalin Township, Chiayi 62247, Taiwan; 9 Department of Medical Research, Dalin Tzu Chi Hospital, Buddhist Tzu Chi Medical Foundation, Chiayi 62247, Taiwan; 10 Department of Environmental and Occupational Health, College of Medicine, National Cheng Kung University, Tainan 333325, Taiwan

**Keywords:** rheumatoid arthritis, rehabilitation services, inflammatory response, generalized estimating equations, follow-up study

## Abstract

*Background and Objectives:* For persons with rheumatoid arthritis (RA), the accompanying systemic inflammatory conditions often insidiously damage extra-glandular organs, causing poor outcomes. Despite evidence manifesting the application of rehabilitation services (RSs), the association between RSs use and changes in the inflammatory response among persons with RA has not yet been established. With that in mind, this study aimed to evaluate changes in C-reactive protein (CRP) and erythrocyte sedimentation rate (ESR) before and after long-term RSs use. *Materials and Methods:* For this two-group pre and posttest study, medical data of 4505 persons with RA aged 20–70 years between 2012 and 2017 were retrieved from an electronic medical record database held by a hospital in Taiwan. Of them, 1387 subjects were categorized as RSs users, who received RSs at least six times within the first year of RA onset. Generalized estimating equations analysis was then employed to compare the changes in ESR and CRP at baseline, and at 12, 18, 24, 30, and 36 months after RA onset. *Results:* After adjusting for inherent differences and mature impact, those receiving standard care plus RSs were found to have a lower CRP level than those without receiving RSs. This benefit was maintained within a 3-year follow-up period. Additionally, a slight but nonsignificant reduction in ESR existed over the same timeframe. *Conclusions:* Integrating RSs into conventional care may be helpful to modulate the inflammation for RA patients, but further research via randomized controlled trials is needed to validate the application of RSs.

## 1. Introduction

Rheumatoid arthritis (RA) is an autoimmune disorder featuring progressive joint destruction resulting from chronic synovial inflammation. RA mainly affects working age people, thus imposing significant consequences on physical functioning and working capacity [1]. One estimate indicated that within the first 3 years after RA onset, approximately 30% of affected people become disabled, thus leading to a socioeconomic burden [2]. According to a recent report, the all-cause societal costs for RA annually is approximately USD 40 billion; likewise, the per-capita healthcare costs for RA patients is USD 20,919, triple that of those without RA [3].

Today, a growing body of evidence supports the notion that the inner inflammation accompanying RA may threaten other organs and systems beyond the joints, and increase risks of cardiovascular disease, pulmonary disease, and cancer [4,5]. In response to inflammation from RA, the body produces C-reactive protein (CRP), and the erythrocyte sedimentation rate (ESR) would increase throughout the body. In clinical practice, therefore, measuring these parameters in the serum can assist to diagnose and monitor related symptoms for persons with RA [6,7]. On top of that, both indicators have been incorporated into disease activity composite scores, including the 28-joint disease activity score (DAS 28). In a study that followed patients with inflammatory arthritis for ten years, it showed that an elevated CRP level (≧5 mg/L) was correlated with a three-fold increase in death due to cardiovascular disease, independent of conventional risk factors [8]. Given that the persistent inflammatory condition significantly worsens outcomes for those afflicted with RA, efforts focusing on breaking the loop of inflammation driven by RA are of paramount importance.

Currently, rehabilitation services (RSs) have been proposed as a potential for restoring RA patients’ functional independence and improving their psychophysical function [7]. The application of RSs in the field of rheumatology mainly covers non-pharmacological therapy and also includes therapeutic patient education, exercises, physical modalities, orthoses, assistive devices, hydrotherapy, and dietary interventions [7]. Former evidence has indicated that hydrotherapy may reduce disease activity, improve mood, and mitigate joint tenderness as compared to no intervention via regulating the inflammatory milieu and oxidative stress [7,9]. Additionally, a recent study of 40 people with RA found that a combined treatment approach of medication and hydrotherapy remarkably abated damage from reactive oxygen species [10]. Some studies using both animal models and human experiments further documented that the use of RSs could be one way to enhance physical function via suppressing the release of bodily inflammatory cytokines, particularly interleukin (IL)-6, IL-1, and tumor necrosis factor-á [11,12], all of which are correlated with the production of acute-phase reactants and rheumatoid cachexia, which impair physical performance [6,7,13].

While there is growing evidence for the benefits of RSs, no concrete, large-scale follow-up data are available to investigate the specific effects of adding RSs into routine care on changes in CRP and ESR in RA patients. A long-term assessment towards this neglected area not only adds to the body of evidence regarding specific therapeutic effects, but also provides novel information for decision makers involved in healthcare services for RA patients. Therefore, to address this gap, we carried out a pretest–posttest follow-up experimental design study to clarify the association between RSs use and changes in CRP and ESR in RA patients.

## 2. Materials and Methods

### 2.1. Research Design and Participants

The retrospective observational study focused on RA patients seen at the rheumatology clinic in a 900-bed regional hospital in Taiwan. Since 2012, the target hospital set up an electronic medical record database (EMRD) to store healthcare claim data, including records of patients’ demographics, diagnoses, prescriptions, and hospitalizations that occurred in the target hospital. The identification numbers of patients in the datasets were encrypted to protect privacy. As the encryption is consistent across all datasets, the encrypted identification numbers remain unique, which makes it feasible to conduct longitudinal follow-up investigations. Prior to commencement, this study was approved by the institutional review board and ethics committee of Buddhist Dalin Tzu Chi Hospital and conducted in accordance with the declaration of Helsinki (No. B11002011).

Herein, we recruited patients aged 20–70 years old who were seen both in outpatient and inpatient settings, diagnosed by the International Classification of Diseases, Ninth Revision, Clinical Modification (ICD-9-CM) with a category of 714.0, from January 2012 to December 2017. The date when each RA patient received the relevant treatments in the target hospital was deemed the index date. In order to improve the accuracy of the RA diagnosis, we only selected RA patients who received treatments from rheumatologists. A total of 6518 RA patients were initially identified for the study. The patients’ relevant disease activity must be evaluated at baseline by their treating rheumatologist and rheumatology nurse and monitored at least every three months thereafter. We excluded persons who did not complete scheduled laboratory tests, or who were followed for less than one year and had incomplete data (n = 2013). A total of 4505 RA subjects were included in the final analysis.

### 2.2. Exposure of Rehabilitation Services Usage

Following the abovementioned filtering process, study participants were further linked to the ambulatory care visit claims to identify their pattern of RSs use after the RA episode. We searched for relevant RSs codes, which comprised 42007A, 42010A9, 42010A7, 42004A2, 42010AD, 42010A5, 42004A8, 42004A, 42010A6, 42004A1, 42016C, 42004A9, 42013A, 42001A, 42013A2, 42013A6, 43026C, 42004A5, 43007A8, 43007A4, 43029A, 43011C, 43010C, 43016C, 43017C, 43007A1, 42107A, 43007AA, 42010A, 44010C, 43015C, 43014C, 42013A1, 44007A2, 44007A9, 44007A, 43007A7, 44007AA, 42004A4, 43007A2, 43012C, 43013C, 42010AB, 44007A8, 43007AF, 43004A, 43007AB, 44004A, 42004A3, and 43007A. In Taiwan, all RSs covered by the National Health Insurance (NHI) are provided only by board-certified physical therapists who received rigorous training in medical schools and through relevant accredited hospital programs. Additionally, the guideline by the NHI indicated that six adjunctive treatments, like acupuncture, had to be delivered every week for six weeks in total to each patient, and are, accordingly, defined as one complete RSs treatment [14]. Accordingly, the participants were identified as RSs users if their records included any of the above codes and had at least six sessions within the timeframe after the onset of RA, and the remaining cases were classified as non-RSs users.

### 2.3. Measurement of Primary Outcomes

**Figure 1:**
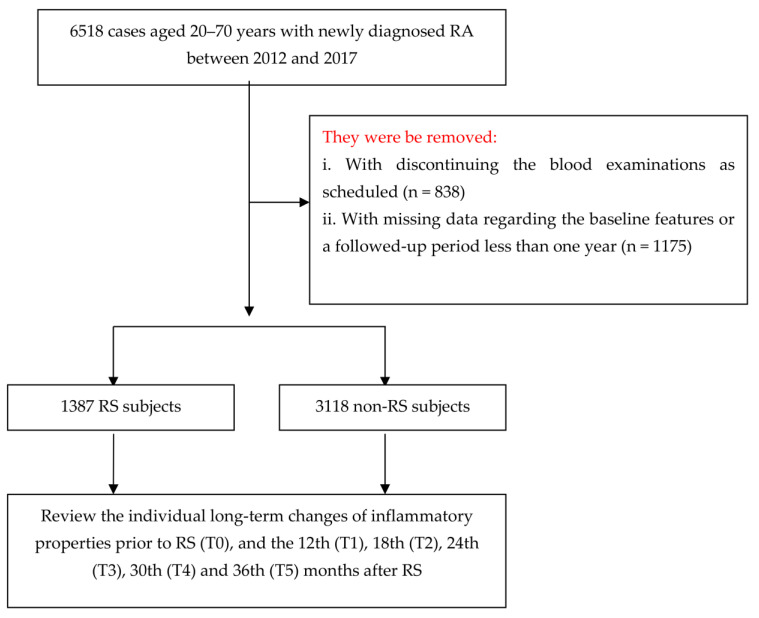
Flowchart showing the method of selecting and following study subjects. RSs, rehabilitation services; RA, rheumatoid arthritis.

Primary outcomes in this work focus on the changes in CRP and ESR. For CRP, the hospital used an automated latex particle-enhanced immunoturbidimetric assay to calculate the CRP level in serum or plasma (Beckman Coulter DxC 700 AU analyzers, Brea, CA, USA). As for ESR, it was measured with quantitative capillary photometry (Sysmex, ESR Analyzer Alifax Test 1, Kobe, Japan). Because the two inflammatory indicators must be regularly monitored for RA patients, we were able to retrieve these records from the EMRD at the following times: baseline (T0), 12 (T1), 18 (T2), 24 (T3), 30 (T4), and 36 (T5) months after the index date (Figure 1).

### 2.4. Covariates

Covariates considered for the study included gender, age, height, body weight, and smoking status [15]. Subjects who reported “currently” or “yes/past” to smoking were classified as users. Comorbid medical conditions for each individual was assessed using the established Charlson–Deyo comorbidity index (CCI) [16]. The CCI contains 17 chronic diseases, with a score between 1 and 6 points, and the sum of these scores is regarded as a measure of comorbidity burden. The comorbidities identified for the CCI included myocardial infarction, congestive heart failure, peripheral vascular disease, cerebrovascular disease, dementia, chronic pulmonary disease, rheumatological disease, peptic ulcer disease, mild liver disease, moderate or severe liver disease, diabetes mellitus (DM), DM with chronic complications, renal diseases, any malignancy, metastatic solid tumor, and HIV infection. To avoid double counting and possible over-adjustment in a regression equation, the diagnosis of RA was excluded from the CCI score in this work. Additionally, medication use was separated into two groups based on whether the patient ever took corticosteroids or disease-modifying anti-rheumatic drugs for more than 6 months after the index date.

### 2.5. Statistical Modeling

We used SAS 9.3 (SAS Institute, Cary, NC, USA) to link the data, and SPSS 22.0 (Chicago, IL, USA) to perform all statistical tests. At the beginning of the analysis, we employed descriptive statistics, including the mean, standard deviation (SD), and percentage, to describe the distributions for participants. Then, the distributions of baseline characteristics for both RSs users and non-RSs users were compared using the Student’s t-test and ÷^2^ test, as applicable. Additionally, intergroup differences before and after RSs involvement, for each of the outcomes, were studied using the generalized estimating equation (GEE) model, which is a statistical procedure that extends the capabilities of generalized linear models for analyzing longitudinal data or other clustered response data [17]. Each GEE model produces estimates in terms of the main effect of time (baseline as the reference category), intervention effect (control group as the reference category), and the effect of one interaction term between the intervention and time after covariate adjustment [18]. The changes in intervention effects over time can then be confirmed from the interaction term providing it was pronounced. Covariates in which the difference reached statistical significance at baseline were regarded as the control variables while using the multivariate GEE model [19]. Robust standard errors were selected to calculate the significance of parameter estimates, and the autoregressive first-order working correlation matrix was utilized to adjust for the time effect [18].

Meanwhile, we also conducted one sensitivity analysis to verify the exposure–response impact of RSs. In this sensitivity analysis, we included the values of ESR and CRP at T0 and T1, and further examined the amplitude of change after separating the use patterns of RSs that occurred within the first year after RA onset, according to their placement in either above or below the 50th percentile of the frequency of RSs use. All statistical significance levels were determined at a two-tailed significance level of less than 0.05.

## 3. Results

### 3.1. Baseline Characteristics for All Participants

Demographic and clinical characteristics of the study sample are given in Table 1. During the study period, a total of 4505 RA patients were recruited, consisting of 1387 in the RSs group and 3118 in the non-RSs group. The mean age of enrollees was 54.2 years. Over half of the enrollees were female and nonsmokers. Significant differences were detected in age, cigarette smoking, weight, and baseline CCI scores between the two groups (all *p* ≤ 0.05).

### 3.2. Comparisons of Levels of CRP and ESR in the RSs Groups Versus Non-RSs Groups

**Figure 2:**
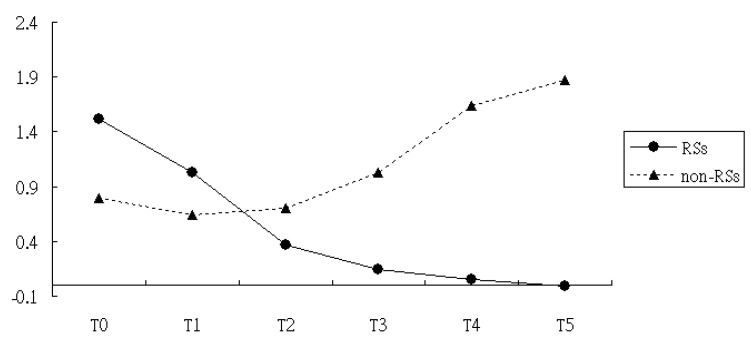
CRP measure over time among patients with and without RSs use. T0, date of first diagnosis of RA; T1, 12 months after RA onset; T2, 18 months after RA onset. T3, 24 months after RA onset; T4, 30 months after RA onset; and T5, 36 months after RA onset.

After adjusting for the variables found to be significant in the univariate analysis, including age, cigarette smoking, weight, and baseline CCI scores, the multivariate analysis with the GEE procedure revealed a baseline difference in the CRP score between the RSs and the non-RSs groups (*p* < 0.01) (Table 2). Meanwhile, CRP levels at T1, T2, T3, T4, and T5 were similar to those measured at T0, implying a nonsignificant maturation effect across follow-up times. After considering the baseline differences of CRP between two groups using the multivariate GEE model, we noted that the reduction slope in the CRP level was still larger in the RSs group compared to the non-RSs group, regardless of the time assessed, with T1, T2, T3, and T4, yielding â = −0.77 (T1), â = −1.25 (T2), â = −2.13 (T3), â = −3.44 (T4), and â = −3.93 (T5), respectively (Table 2 and Figure 2).

**Figure 3:**
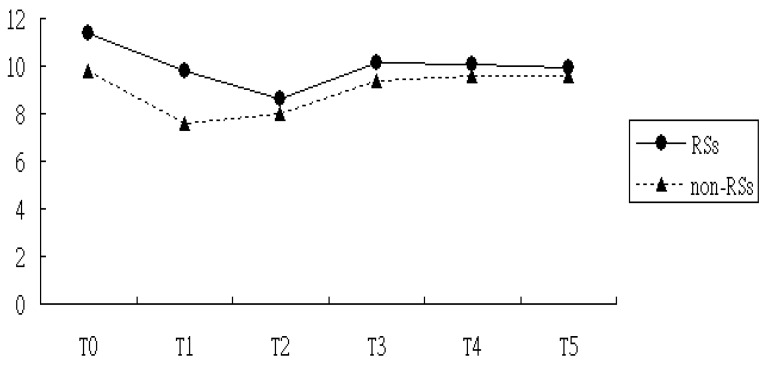
ESR measure over time among patients with and without RSs use. T0, date of first diagnosis of RA; T1, 12 months after RA onset; T2, 18 months after RA onset; T3, 24 months after RA onset; T4, 30 months after RA onset; and T5, 36 months after RA onset.

For ESR, the GEE model reported that a baseline difference existed between the group treated with RSs and the nontreated group, supporting estimates calculated from the Student’s t-test. On top of that, the ESR levels at T1 and T2 were remarkably different from those at T0, implying a maturation effect might have emerged. In other words, the mean ESR level at T1 and T2 was significantly lower than the mean ESR at T0, even without the RSs intervention. After corrections for baseline differences along with the maturation effect, we found that the reduction in ESR was larger in the RSs group than the non-RSs group during the study timeframe, although the difference did not reach statistical significance (Table 2 and Figure 3).

After carrying out a sensitivity analysis, where only the values of ESR and CRP at T0 and T1 among subjects were used, it was revealed that RSs use intensity was inversely correlated with CRP level, but not for ESR (Table 3). In a nutshell, for RA subjects, the more frequent usage of RSs was associated with a lower the CRP level after RA onset.

## 4. Discussion

RSs use has long been a frequently employed complementary approach for the management of RA. Despite this, our current understanding of the relationships between RSs application and changes in inflammatory indicators is still lacking, and previous studies have either used small samples or have been cross-sectional in nature [9,10]. To our knowledge, this is the first study to directly examine the relationships between RSs use and the subsequent changes in inflammatory properties among RA persons. Over the 3-year follow-up period, we noted that the integration of RSs to standard care did ameliorate inflammation in RA patients, especially as measured by CRP levels. The merit of this study is that we capitalized on empirical research using follow-up measures, which allowed us to deliberately clarify the potential causal relationship [20]. This provides for robust and empirically informed guidance for healthcare providers while managing RA patients.

After fitting the GEE multivariate model on the basis of the first-order autoregressive procedure, we found that integrating RSs into routine care substantially reduced CRP levels, supporting previous research, which found that receiving 3-month hydrotherapy sessions in addition to conventional treatment substantially reduced levels of reactive oxygen species among RA patients [10]. We speculated that direct participation in the relevant RSs programs may simultaneously provide intangible benefits as well, such as allowing patients to socialize and share joyous moments during RSs sessions, which in turn may reduce stress, and consequently decrease the expression of serum inflammatory mediators like IL-6, IL-1, and tumor necrosis factor-á [11,21]. In a prior murine model with collagen-induced arthritis, the authors observed that the rats, which were placed on a treadmill to exercise for 15 min per day for three weeks, had markedly reduced levels of inflammatory factors, thus improving the inflammation-related joint damage via the suppression of NFATc1and NF-êB luciferase activities [12]. It is well known that inflammatory markers are deeply involved in the production of CRP in hepatocytes [6,7]. Since downregulated NF-êB expression has been implicated in various inflammatory diseases [7,22], instituting several novel RSs approaches targeting this intracellular pathway may be considered, especially in the management of patients with rheumatic diseases.

Unlike the positive impact of RSs on CRP, our findings indicated a recognizable, yet not statistically significant, decrease in the ESR in the RSs group. In clinical practice, these two acute phase reactants are frequently used as markers for clinical signs and symptoms of inflammation. We speculate that the ESR measure is more likely to be influenced by the concentrations of fibrinogen and alpha globulins [23]. Fibrinogen is a fibrous protein known to be a slow-reacting positive acute phase reactant [24]. Therefore, ESR is relatively insensitive to the inflammatory process, especially for patients with systemic rheumatic diseases and those with concomitant renal insufficiency, low albumin, and anemia [25,26]. Thus, of studies conducted thus far, CRP has been acknowledged to be a better indicator of the acute inflammation phase, and has yielded a sensitivity of 0.86, specificity of 0.67, and summary receiver operating characteristic curve (SROC) of 0.86, as compared with a sensitivity of 0.77, specificity of 0.59, and SROC of 0.75 for ESR [27]. This, in a nutshell, is the reason that a nonsignificant ESR reduction was observed for the RSs group.

### Limitations

The GEE approach, an enlargement of the generalized linear model approach, can model the correlated data via the introduction of second-order variance components. To our knowledge, this was the first evidence-based longitudinal study to explore the association between RSs use and changes in CRP and ESR among RA patients after fitting the GEE procedure, thereby allowing us to clarify the magnitude of RSs effect. While considerable efforts were made in the design of this study, there were several drawbacks related to the sample selection process, ability to generalize findings, and research design. First and foremost, patients from a single center were enrolled; therefore, our results may not generalize to groups with different demographic and geographic features. Additional research focusing on more targets should be considered in heterogeneous populations to confirm the replicability of present findings. Second, since all analytical data were extracted from a claims-based database, several key factors, such as lifestyle behaviors, family history, and biochemical data, were not available. To minimize the effect of confounding during the statistical analysis, we set objective criteria to balance differences between groups treated or not treated with RSs via assessments of demographic and disease variables, as shown in Table 1. In addition to utilizing multivariate analysis to control for inherent baseline differences between patients, we further considered the potential maturation effect via the GEE procedure, thus minimizing the probability of an inflated type I error [18]. Third, the related RSs received during treatment were integrated in one consolidated column of the database. In such a case, we merely abstracted the corresponding fees from this to ascertain the RSs exposure and were unable to clarify the specific RSs type. Future works, through the applications of various recruitment methods in ethnically diverse populations*,* are recommended. Fourth, although our study revealed a substantial beneficial association between RSs use and a decreased CRP level among RA patients, it must be recognized that participants were not initially randomly categorized into users and nonusers and were recruited from a single country only. Even so, preceding the RSs intervention, we observed that RSs subjects possessed higher levels of CCI, CRP, and ESR than did non-RSs users at the index date (Table 1), which implies that the conclusions reported would underestimate, rather than overestimate, the effects of RSs. That being said, a study adopting a larger cohort of RA patients using a randomized clinical trial design is still needed to minimize confounding by covariates not explicitly accounted for in the present design.

## 5. Conclusions

Faced with the dire harmful manifestations caused by inflammation, it is of importance to extend the horizons of traditional therapy procedures and explore the role of RSs in alleviating inflammation among RA patients. The findings of the present study suggest that integrating RSs into standard care may partially lower the subsequent systemic inflammation response, especially in CRP levels. This benefit is sustained over a 3-year follow-up period. Our findings support the integration of RSs into routine disease management for those with rheumatological disorders. This study not only bridges the chasm regarding the long-term assessment of RSs usage to changes in inflammatory parameters, but also provides an impetus for further in vivo research that focuses on the exploration of the beneficial mechanisms of RSs in the treatment of chronic diseases. Therefore, we recommend that healthcare providers proactively assess patients’ inflammatory status and implement this novel and safe regimen in managing RA.

## Figures and Tables

**Table 1:** Demographic and clinical characteristics of study participants by group.

Variables	All Enrollees (*n* = 4505)		RSs Group (*n* = 1387)		Non-RSs Group (*n* = 3118)		*p*
	N	%	N	%	N	%	
Demographic data							
Sex							<0.01
Female	3443	76.4	1109	80.0	2334	74.9	
Male	1062	23.6	278	20.0	784	25.1	
Cigarette smoking							0.001
Yes	63	1.4	31	2.2	32	1.0	
NO	4442	98.6	1356	97.8	3086	99.0	
Age (mean ± SD)	54.2 ± 13.1		56.2 ± 12.7		53.25 ± 13.1		<0.01
Height	156.6 ± 7.4		156.9 ± 7.6		156.3 ± 7.3		0.65
Weight	59.9 ± 10.9		62.7 ± 11.3		57.6 ± 10.0		0.01
Clinical characteristics							
Medication use							0.06
Yes	3090	68.6	923	66.5	2167	69.5	
No	1415	31.4	464	33.5	951	30.5	
CCI (mean ± SD)	2.56 ± 1.32		3.17 ± 1.34		2.29 ± 1.30		<0.01
Baseline CRP (mean ± SD)	1.14 ± 2.91		1.63 ± 3.38		0.92 ± 2.13		<0.01
Baseline ESP (mean ± SD)	20.37 ± 11.42		21.46 ± 14.10		19.86 ± 10.02		0.03

SD, standard deviation; CCI, Charlson–Deyo comorbidity index; CRP, C-reactive protein; ESR, erythrocyte sedimentation rate; RSs, rehabilitation services.

**Table 2:** Effects of RSs on inflammation level in the participants by GEE model.

Variables	CRP		ESR	
	Regression Coefficient ^+^	*p*	Regression Coefficient ^+^	*p*
Intercept	0.79	<0.01	9.80	<0.01
RSs vs. non-RSs	0.73	<0.01	1.56	0.04
T1 vs. T0	−0.15	0.07	−4.0	<0.01
T2 vs. T0	−0.09	0.10	−3.83	0.02
T3 vs. T0	0.24	0.22	−1.39	0.14
T4 vs. T0	0.85	0.09	−0.89	0.49
T5 vs. T0	1.08	0.06	−0.41	0.16
Interaction of T1×Group	−0.22	0.04	1.81	0.22
Interaction of T2×Group	−1.06	0.01	1.09	0.18
Interaction of T3×Group	−2.13	<0.01	0.27	0.14
Interaction of T4×Group	−3.02	<0.01	−0.11	0.08
Interaction of T5×Group	−2.72	<0.01	−0.20	0.07

RSs, rehabilitation services; T0, date of first diagnosis of RA; T1, 12 months after RA onset; T2, 18 months after RA onset. T3, 24 months after RA onset. T4, 30 months after RA onset. T5, 36 months after RA onset. RSs vs. non-RSs represents the baseline difference between two groups. T1 vs. T0, change between T0 and T1; T2 vs. T0, change between T0 and T2; T3 vs. T0, change between T0 and T3; T4 vs. T0, change between T0 and T4; T5 vs. T0, change between T0 and T5. Interaction of T1×Group, difference between RSs and non-RSs groups within the period of T0–T1; Interaction of T2×Group, difference between RSs and non-RSs groups within the period of T0–T2; Interaction of T3×Group, difference between RSs and non-RSs groups within the period of T0–T3; Interaction of T4×Group, difference between RSs and non-RSs groups within the period of T0–T4; and Interaction of T5×Group, difference between RSs and non-RSs groups within the period of T0–T5. **^+^** adjustment for age, cigarette smoking, weight, and baseline CCI scores.

**Table 3:** Sensitivity analysis regarding the effect of RSs on inflammation properties by different usage pattern.

RSs Usage Pattern	CRP		ESR	
	Regression Coefficient	*p*	Regression Coefficient	*p*
Non-RSs group	1		1	
RSs group	−0.54	<0.01	1.33	0.52
Low intensity	−0.37	0.003	1.74	0.29
High intensity	−0.69	<0.01	1.26	0.08

CRP, C-reactive protein; ESR, erythrocyte sedimentation rate; RSs, rehabilitation services.

## Data Availability

The data presented in this study are available on request from the corresponding author.

## References

[1] Kirkeskov L., Bray K. (2023). Employment of patients with rheumatoid arthritis—A systematic review and meta-analysis. BMC Rheumatol..

[2] Sokka T. (2003). Work disability in early rheumatoid arthritis *Clin*. Exp. Rheumatol..

[3] Chen C.I., Wang L., Wei W., Yuce H., Phillips K. (2018). Burden of rheumatoid arthritis among US medicare population: Co-morbidities, health-care resource utilization and costs. Rheumatol. Adv. Pract..

[4] Burska A., Boissinot M., Ponchel F. (2014). Cytokines as biomarkers in rheumatoid arthritis. Mediat. Inflamm..

[5] Cook M.J., Bellou E., Bowes J., Sergeant J.C., O’Neill T.W., Barton A., Verstappen S.M.M. (2018). The prevalence of co-morbidities and their impact on physical activity in people with inflammatory rheumatic diseases compared with the general population: Results from the UK Biobank. Rheumatology.

[6] Pope J.E., Choy E.H. (2021). C-reactive protein and implications in rheumatoid arthritis and associated comorbidities. Semin. Arthritis Rheum..

[7] D’Cruz L.G., McEleney K.G., Cochrane C., Tan K.B., Shukla P., Gardiner P.V., Small D., Zhang S.D., Gibson D.S. (2020). Assessment of a dried blood spot C-reactive protein method to identify disease flares in rheumatoid arthritis patients. Sci. Rep..

[8] Goodson N.J., Symmons D.P., Scott D.G., Bunn D., Lunt M., Silman A.J. (2005). Baseline levels of c-reactive protein and prediction of death from cardiovascular disease in patients with inflammatory polyarthritis: A ten-year follow-up study of a primary care–based inception cohort. Arthritis Rheum..

[9] Al-Qubaeissy K.Y., Fatoye F.A., Goodwin P.C., Yohannes A.M. (2013). The effectiveness of hydrotherapy in the management of rheumatoid arthritis: A systematic review. Musculoskelet. Care.

[10] Mateen S., Moin S., Khan A.Q., Zafar A., Fatima N., Shahzad S. (2018). Role of hydrotherapy in the amelioration of oxidant-antioxidant status in rheumatoid arthritis patients. Int. J. Rheum. Dis..

[11] Gleeson M., Bishop N.C., Stensel D.J., Lindley M.R., Mastana S.S., Nimmo M.A. (2011). The anti-inflammatory effects of exercise: Mechanisms and implications for the prevention and treatment of disease. Nat. Rev. Immunol..

[12] González-Chávez S.A., López-Loeza S.M., Acosta-Jiménez S., Cuevas-Martínez R., Pacheco-Silva C., Chaparro-Barrera E., Pacheco-Tena C. (2023). Low-intensity physical exercise decreases inflammation and joint damage in the preclinical phase of a rheumatoid arthritis murine model. Biomolecules.

[13] Kohn L.T., Corrigan J.M., Donaldson M.S. (2000). To Err is Human: Building a Safer Health System.

[14] Lu M.C., Livneh H., Yen C.T., Huang H.L., Lin M.C., Yen S.W., Lai N.S., Tsai T.Y. (2020). Association of use of rehabilitation services with development of dementia among patients with rheumatoid arthritis: Analysis of domestic data in Taiwan. Front. Med..

[15] Aletaha D., Smolen J.S. (2018). Diagnosis and management of rheumatoid arthritis: A review. JAMA.

[16] Deyo R.A., Cherkin D.C., Ciol M.A. (1992). Adapting a clinical comorbidity index for use with ICD-9-CM administrative databases. J. Clin. Epidemiol..

[17] Zeger S.L., Liang K.Y. (1986). Longitudinal data analysis for discrete and continuous outcomes. Biometrics.

[18] Overall J.E., Tonidandel S. (2004). Robustness of Generalized Estimating Equation (GEE) tests of significance against misspecification of the error structure model. Biom. J..

[19] Lu M.-C., Guo H.-R., Livneh H., Lin M.-C., Lai N.-S., Tsai T.-Y. (2020). The effectiveness of nurse-led case management for patients with rheumatoid arthritis in Taiwan. Int. J. Clin. Pract..

[20] Letnes J.M., Nes B.M., Wisløff U. (2023). Age-related decline in peak oxygen uptake: Cross-sectional vs. longitudinal findings. A review. Int. J. Cardiol. Cardiovasc. Risk Prev..

[21] Kelley G.A., Kelley K.S., Hootman J.M. (2015). Effects of exercise on depression in adults with arthritis: A systematic review with meta-analysis of randomized controlled trials. Arthritis Res. Ther..

[22] Qu Z., Zhang B., Kong L., Gong Y., Feng M., Gao X., Wang D., Yan L. (2022). Receptor activator of nuclear factor-IoB ligand-mediated osteoclastogenesis signaling pathway and related therapeutic natural compounds. Front. Pharmacol..

[23] Litao M.K., Kamat D. (2014). Erythrocyte sedimentation rate and C-reactive protein: How best to use them in clinical practice. Pediatr. Ann..

[24] Doolittle R.F., Malagoli D. (2016). Chapter 11—Structural and Functional Diversity of Fibrinogen-Related Domains. The Evolution of the Immune System.

[25] Harrison M. (2015). Erythrocyte sedimentation rate and C-reactive protein. Aust. Prescr..

[26] Costenbader K.H., Chibnik L.B., Schur P.H. (2007). Discordance between erythrocyte sedimentation rate and C-reactive protein measurements: Clinical significance. Clin. Exp. Rheumatol..

[27] Lapiæ I., Padoan A., Bozzato D., Plebani M. (2020). Erythrocyte sedimentation rate and c-reactive protein in acute inflammation. Am. J. Clin. Pathol..

